# Women and Men Differ in Relative Strengths in Wisdom Profiles: A Study of 659 Adults Across the Lifespan

**DOI:** 10.3389/fpsyg.2021.769294

**Published:** 2022-02-03

**Authors:** Emily B. H. Treichler, Barton W. Palmer, Tsung-Chin Wu, Michael L. Thomas, Xin M. Tu, Rebecca Daly, Ellen E. Lee, Dilip V. Jeste

**Affiliations:** ^1^VA Desert Pacific Mental Illness Research, Education, and Clinical Center (MIRECC), San Diego, CA, United States; ^2^Department of Psychiatry, University of California, San Diego, San Diego, CA, United States; ^3^Sam and Rose Stein Institute for Research on Aging, University of California, San Diego, San Diego, CA, United States; ^4^VA San Diego Healthcare System, San Diego, CA, United States; ^5^Department of Family Medicine and Public Health, University of California, San Diego, San Diego, CA, United States; ^6^Department of Psychology, Colorado State University, Fort Collins, CO, United States; ^7^Department of Neurosciences, University of California, San Diego, San Diego, CA, United States

**Keywords:** age, positive psychiatry, compassion, self-reflection, emotional regulation

## Abstract

Wisdom is a multi-component trait that is important for mental health and well-being. In this study, we sought to understand gender differences in relative strengths in wisdom. A total of 659 individuals aged 27–103 years completed surveys including the 3-Dimensional Wisdom Scale (3D-WS) and the San Diego Wisdom Scale (SD-WISE). Analyses assessed gender differences in wisdom and gender’s moderating effect on the relationship between wisdom and associated constructs including depression, loneliness, well-being, optimism, and resilience. Women scored higher on average on the 3D-WS but not on the SD-WISE. Women scored higher on compassion-related domains and on SD-WISE Self-Reflection. Men scored higher on cognitive-related domains and on SD-WISE Emotion Regulation. There was no impact of gender on the relationships between wisdom and associated constructs. Women and men have different relative strengths in wisdom, likely driven by sociocultural and biological factors. Tailoring wisdom interventions to individuals based on their profiles is an important next step.

## Introduction

Wisdom is one of six core virtues shared across cultures (Peterson and Seligman, 2004; Dahlsgaard et al., 2005), and an increasing area of interest in mental health disciplines due to its link with health, mental health, and well-being (Staudinger and Glück, 2011; Webster et al., 2014; Jeste et al., 2020b). Although the study of wisdom was developed and nurtured in the humanities, in recent decades psychology, psychiatry, and related disciplines have begun to study the topic empirically (Bangen et al., 2013; Glück, 2020; Jeste et al., 2020b). Wisdom is a complex, multi-component trait that includes cognitive, reflective, and affective dimensions (Ardelt, 2003). There are several components common across many proposed definitions of wisdom in the literature – viz., pro-social behaviors and attitudes, including empathy and compassion; emotional regulation, with a tendency toward stable, positive mood; self-reflection and awareness; ability to acknowledge and tolerate uncertainty and disagreement; decisiveness; and social advising (Meeks and Jeste, 2009). Some experts also consider spirituality to be a component of wisdom, although there is less consensus on the latter conclusion (Jeste et al., 2020c). Therefore, the contemporary empirical model of wisdom includes and integrates pragmatic elements, like effective social and decision-making skills, and broader elements, like acceptance of self and others, that harken back to its philosophical origins.

The burgeoning positive psychiatry subfield focuses on improving outcomes like quality of life and well-being (Jeste et al., 2015), so wisdom has found a natural home there. Similarly to related domains in positive psychiatry such as resilience and optimism, there is evidence that it is possible to increase wisdom, and that doing so also increasing in quality of life and well-being (Kim et al., 2013; McLaughlin et al., 2018; Paredes et al., 2020; Treichler et al., 2020). A recent meta-analysis of randomized controlled trials targeting individual components of wisdom including empathy and compassion found that nearly one-half of the psychosocial or behavioral interventions were effective with medium to large effect sizes (Lee et al., 2020). Therefore, determining differences in levels of wisdom and its components in specific subgroups of people would help target and tailor associated interventions both at the individual and public health level. For example, one study of emerging adults found four distinct wisdom profiles (Booker and Dunsmore, 2016) indicating different levels of need for intervention, and areas of focus for that potential intervention. More broadly, use of wisdom assessment in clinical practice might be useful in identifying which of existing interventions might be the best fit.

In this article, we examine the association between gender and wisdom. Some reviews and theoretical work have pronounced wisdom should be “androgynous” (Aldwin, 2009) and emphasized wisdom constructs must actively avoid gender bias (Levenson, 2009) both in terms of their conceptualization and their measurement. Yet, of the six studies we found in a thorough but non-systematic review of the literature focused on gender differences in wisdom (Ardelt, 2009; Kanwar, 2013; Singh and Dahiya, 2013; Bang, 2015; Cheraghi et al., 2015; Maroof et al., 2015), and the nine additional studies that included secondary or exploratory analyses about gender differences in wisdom (Ardelt, 1997, 2015; Yang, 2001; Webster, 2003; Levenson et al., 2005; Glück et al., 2013; Bang and Zhou, 2014; König and Glück, 2014; Brienza et al., 2018), nine reported gender differences in one or more analysis (Ardelt, 2003, 2009; Webster, 2003; Kanwar, 2013; Singh and Dahiya, 2013; Bang and Zhou, 2014; Cheraghi et al., 2015; Maroof et al., 2015; Brienza et al., 2018). The other six studies did not find gender differences in any analysis.

The direction of the differences identified were mixed. In three studies of the nine studies where gender differences were found, women scored higher on overall wisdom (Webster, 2003; Singh and Dahiya, 2013; Bang and Zhou, 2014). The rest of the studies did not find an overall pattern where one gender scored higher than the other, but rather found differences in one or more subdomains. These subdomain findings as a whole do not create a clear pattern. For example, one study found that women scored higher on the affective or compassionate subscale (Ardelt, 2009) while another found that men scored higher on the same subscale (Maroof et al., 2015). Studies have reported a variety of findings regarding gender differences in the cognitive subdomain, including that women of all ages score lower (Ardelt, 2003), only older women score lower (Cheraghi et al., 2015), and only older men score higher (Ardelt, 2009). Notably, these studies varied in the mean age of the sample, the sample size, the measure(s) of wisdom and well-being used, and the country or region where the data were collected, which also relates to the variability in race and ethnicity of the sample, and the cultural values of the sample. These differences may impact the findings themselves; study found that men in a non-North American scored higher at wise reasoning in conflict than non-North American women, but there were no differences between North American men and women in wise reasoning in conflict, or between women and men in either group for any other subdomain (Brienza et al., 2018). Which conceptualization of wisdom is being measured also plays a role: a study in India using a less frequently used measure found that men scored higher only on the humor subscale (Kanwar, 2013).

Examination of related psychological constructs yields support for gender differences in a subset of wisdom components. A meta-analysis of the 24 character traits making up the six virtues, including wisdom, found differences between women and men in 17 traits, although 13 of these were very small in size (Heintz et al., 2019). There were small to medium effect sizes in appreciation of beauty/excellence, gratitude, kindness, and love, with women scoring higher in each category on average. Evidence for gender differences in emotional regulation is mixed, broadly indicating that women and men utilize different emotional regulation strategies and the choice of strategy impacts mental health outcomes (Nolen-Hoeksema and Aldao, 2011; Nolen-Hoeksema, 2012; Zimmermann and Iwanski, 2014). Women have been consistently reported to have higher empathy and compassion toward others (e.g., Eisenberg and Lennon, 1983; Sprecher and Fehr, 2005; Martins et al., 2013; Pommier et al., 2020). Compassion is the single most important component of wisdom, providing more evidence that wisdom varies by gender.

Gender norms, differential approaches to upbringing and socialization, and other sociocultural factors may support increased development of some areas of wisdom in women compared to men and vice versa. Related work finds that women and men report some variance in terms of how they conceptualize wisdom, where women are somewhat more likely to endorse a “integrative” model while men are somewhat more likely to endorse a “cognitive” model (König and Glück, 2013). This variance may impact the development of wisdom. For example, an international study of 800 female and male adolescents 15–18 years old identified different conceptualizations of wisdom; male adolescents tended to describe wise people as calculating, strict, and questioning, while female adolescents tended to conceptualize wise people as cooperative, optimistic, extroverted, and spontaneous (Hollingworth et al., 2013). However, gender differences between adults in wisdom concepts tend to be quite small (e.g., Glück et al., 2009; Glück and Bluck, 2011; König and Glück, 2013), although they do follow the integrative/cognitive pattern. Women are more likely to rate confronting mortality and dealing with negative life events as relevant to developing wisdom, while men were more likely to think that studying philosophy was relevant (Glück et al., 2009). Similarly, when women and men with significant work histories are asked to report situations where they have been wise, 44.9% of men report work experiences, while only 21.2% of women report work experiences, and are more likely to report a negative experience including a loss or death (Glück et al., 2009). Therefore, although there is notable overlap in wisdom conceptualization across gender, there are also some distinctions. Those distinctions may translate into differences into how people develop or intentionally pursue wisdom (Hollingworth et al., 2013). Ardelt (2003) also suggested that women and men may receive different levels of support from others in the pursuit of different components of wisdom based on gender-based norms; i.e., that an average man might be encouraged to advance their cognitive wisdom through learning and reflection more than an average woman. This idea is supported by evidence that some components of wisdom tend to be societally seen as a “belonging” to one gender, for example, decisiveness is often seen as a masculine trait (Alexander and Andersen, 1993; Ronk, 1993; Hofstede, 1996). This could mean that a woman and a man with baseline equal levels of wisdom, or wisdom capabilities, might have different levels of wisdom cultivation overall, and particularly by subarea due to internal and external factors, both influenced by sociocultural gender norms. Although we expect that sociocultural factors make up the majority of any gender differences in wisdom, there are also biological underpinnings to wisdom (Meeks and Jeste, 2009) which are likely to vary based on different aspects of biological sex (e.g., hormonal variance). Although gender and sex are not the same construct, gender groups show biologically based variances in some ways (Rametti et al., 2011; Hines, 2020; Kiyar et al., 2020).

Thus, the existing work in this area argues for a gender-neutral wisdom construct, but the empirical studies show mixed findings regarding the current gender-based differences in wisdom, which may exist for sociocultural and biological reasons. Identifying and characterizing gender differences is important because it will better illuminate the current wisdom construct as it is being commonly measured today, and whether it differs from the gender-neutral goal originally sought. It also allows for the consideration of individualized pathways to wisdom. The existence of wisdom subdomains, alongside previous discussion regarding individual development and pursuit of wisdom (e.g., Aldwin, 2009; Hollingworth et al., 2013), suggests the existence of ‘wisdom profiles,’ with varying strengths and weaknesses. It may further suggest that individuals, and perhaps identifiable groups like women and men, may pursue and achieve wisdom and its associated positive psychiatry outcomes, via different paths. Identifying and deeply understanding these groups, profiles, and pathways will facilitate improved understanding of the wisdom concept itself as well as how support development of wisdom and promotion of associated outcomes in positive psychiatry. The growing set of positive psychiatry interventions often target wisdom and its subdomains alongside other related outcomes, like resilience and optimism, and sometimes also intend to improve symptoms, emotional distress, or social disconnectedness (e.g., Lee et al., 2020). This approach may include implicit assumptions that these constructs are linearly associated with each other across individuals and groups. However, given the possibility of “wisdom profiles,” that vary among groups like women and men, understanding how those profiles impact the relationship between wisdom and associated constructs targeted in positive psychiatry interventions would be useful for ensuring that these interventions are maximally effective at the group and individual level.

### Aims and Hypotheses

Therefore, in this study, we examined gender differences in a relatively large community-based sample across the adult lifespan using two validated rating scales (Ardelt, 2003; Thomas et al., 2019). We included other relevant measures related to well-being to better understand whether any potential differences in wisdom between women and men impacted the relationship between wisdom and those constructs associated with well-being.

Based on the existing literature, our first hypothesis was that compassion-related domains such as the Affective or Compassionate dimension of the 3-Dimensional Wisdom Scale (3D-WS), and Pro-Social Behaviors and Acceptance of Diverse Perspectives on the San Diego Wisdom Scale would be higher among women. The second hypothesis was that men would score higher on cognitive-related domains like the Cognitive dimension of the 3D-WS and Decisiveness on the San Diego Wisdom Scale. The third hypothesis was that women would score higher on wisdom total scores. Given significant differences between women and men in this same sample in age, income, education, and marital status, these variables were also included in the model. We conducted two sets of exploratory analyses: first, to test whether the magnitude of gender differences in wisdom would vary between more wise and less wise individuals, as posited by Orwoll and Achenbaum (1993) and supported by Ardelt (2009); and second, to test whether gender impacted the relationship between wisdom and measures of well-being, to understand whether potential gender differences might impact how wisdom relates to other constructs typically targeted by positive psychiatry interventions.

## Materials and Methods

This study was approved by the University of California, San Diego Human Research Protections Program (#171635).

### Participants

The participants in this study were recruited from the UCSD Successful Aging Evaluation (SAGE) study, an ongoing project which has been described in previous work (Thomas et al., 2016). Briefly, SAGE is a multicohort study targeting adults across the lifespan. Participants included adults aged 27–103 who are currently living in the community, physically and mentally able to complete the assessments, have access to a phone in their homes, and have no known dementia diagnosis. People living in nursing homes, those who required daily skilled nursing care, and those with a terminal illness were excluded. The study oversampled adults over age 75 because adults in this age group tend to be under-represented in studies of aging. Participants were identified through random digit dialing and completed a 25-min initial phone interview followed by a survey that was mailed or completed online. Participants were provided information about the study in a packet to guide them in their decision to participate; however, documented consent was waived by the human protections review board. In this study, we used the assessment done in 2018 or 2019, when we had data on both the scales of wisdom, creating a cross-sectional dataset of 659 individuals.

### Measures

Two measures of wisdom with good to excellent psychometric properties including reliability, convergent validity, and divergent validity were included in this study. The first was the 39-item 3D-WS, which has three subscales capturing the Cognitive, Reflective, and Affective (Compassionate) dimensions of wisdom (Ardelt, 2003). The second measure was the 24-item San Diego Wisdom Scale (SD-WISE) (Thomas et al., 2019) which has six subscales: Social Advising, Decisiveness, Emotional Regulation, Self-Reflection (previously called Insight), Acceptance of Diverse Perspectives (previously called Tolerance for Divergent Values), and Pro-Social Behaviors.

Gender was self-reported with two categorical options: “male” or “female.” This approach allowed for examining differences between people identifying as men and women, the two most frequently reported genders (Richards et al., 2016), but has inherent limitations because it does not apply to all people’s experience of their gender (Hyde et al., 2019).

Additional measures were included to examine associated constructs: the Center for Epidemiologic Studies Depression Scale (CES-D) (Radloff, 1977), UCLA Loneliness Scale – Third Edition (ULS) (Russell, 1996), The Mental Wellbeing subscale of the SF-36 (Ware, 2000), Life Orientation Test-Revised (Glaesmer et al., 2012) (LOT-R, a measure of optimism), and Connor Davidson Resilience Scale (Campbell-Sills and Stein, 2007) (CD-RISC).

### Statistical Analysis

Linear models were performed to examine the relationship between wisdom (the dependent variable) and gender (independent variable). Income, education, age, and marital status were also included as covariates because there were significant differences between women and men in this sample on those demographic areas. One model was calculated for 3D-WS total score, SD-WISE total score, and subscale scores for each measure. Cohen’s *d* was calculated for each gender effect. For the first set of exploratory analyses, a median split was calculated for 3D-WS total score, SD-WISE total score, and subscale scores for each measure. Separate linear models were performed to identify how the interaction between gender and the median split dummy coded variable impacted the relationship between gender and each wisdom variable. For the second set of exploratory analyses, separate linear models were performed to identify how the interaction between gender and each wisdom total score and subscale score impacted CES-D (Depression), the UCLA-3 Loneliness, SF-36 Mental Well-being, LOT-R Optimism, and CD-RISC Resilience scores. These analyses were adjusted for family-wise error by using the false discovery rate (FDR) correction.

## Results

Detailed demographic comparison of men and women in the sample is presented in Table 1.

**TABLE 1:** Demographic and clinical characteristics of women and men.

	Women				Men				
	*N*	Mean or %	SD	Range	*N*	Mean or %	SD	Range	*p*
**Sociodemographic**									
Age (years)	334	64.76	19.8	27.35–103.78	325	69.0	18.31	27.36–102.67	**0.004**
Race (% white)	244	73.1			253	77.8			0.49
Education (%)									**0.03**
High School and Below	34	10.18			25	7.7			
Some College to Bachelor’s Degree	181	54.19			162	49.85			
Post-Graduate Degree	117	35.03			135	41.54			
Household Income (%)									**0.001**
<$35,000	56	16.77			27	8.31			
$35,000 – $74,999	61	18.26			59	18.15			
$75,000+	160	47.90			190	58.46			
Current Marital Status (% marriage-like)	160	48.20			246	76.16			**<0.001**
**Wisdom Measures**									
3-Dimensional Wisdom Scale (3D-WS)	310	3.69	0.43		314	3.56	0.45		**0.01**
**3D-WS Cognitive Dimension**	310	3.55	0.50		314	3.47	0.56		**0.019**
**3D-WS Reflective Dimension**	310	3.91	0.55		314	3.88	0.56		0.94
**3D-WS Affective (Compassionate) Dimension**	310	3.60	0.51		314	3.56	0.53		**0.008**
San Diego Wisdom Scale (SD-WISE)	310	3.86	0.43		314	3.86	0.45		0.55
**SD-WISE Social Advising**	310	3.81	0.55		314	3.71	0.62		**0.02**
**SD-WISE Decisiveness**	310	3.61	0.81		314	3.83	0.70		**0.015**
**SD-WISE Emotional Regulation**	310	3.58	0.69		314	3.80	0.64		**<0.001**
**SD-WISE Self-Reflection**	310	3.85	0.62		314	3.73	0.65		**0.03**
**SD-WISE Acceptance of Diverse Perspectives**	310	4.05	0.55		314	3.89	0.62		**0.001**
**SD-WISE Pro-Social Behaviors**	310	4.28	0.49			4.23	0.50		**0.035**
**Wellbeing Measures**									
Depression (CES-D)	319	5.56	5.27		315	5.10	4.55		0.24
Resilience (CD-RISC)	329	29.94	6.09		320	31.36	6.17		**0.03**
Optimism (LOT-R)	319	23.71	4.14		317	23.71	3.96		0.64
Loneliness (ULS)	315	36.33	10.44		314	36.61	10.18		0.74
Mental Wellbeing (SF-36)	326	52.90	9.21		322	54.07	8.32		0.09

*All Wisdom p-values corrected as described in sections “Materials and Methods” and “Results.” CES-D, Center for Epidemiological Studies Depression Scale; CD-RISC, Connor-Davidson Resilience Scale; LOT-R, Life Orientation Task-Revised; ULS, UCLA Loneliness Scale. Bolded p values are significant; p < 0.05.*

**FIGURE 1:**
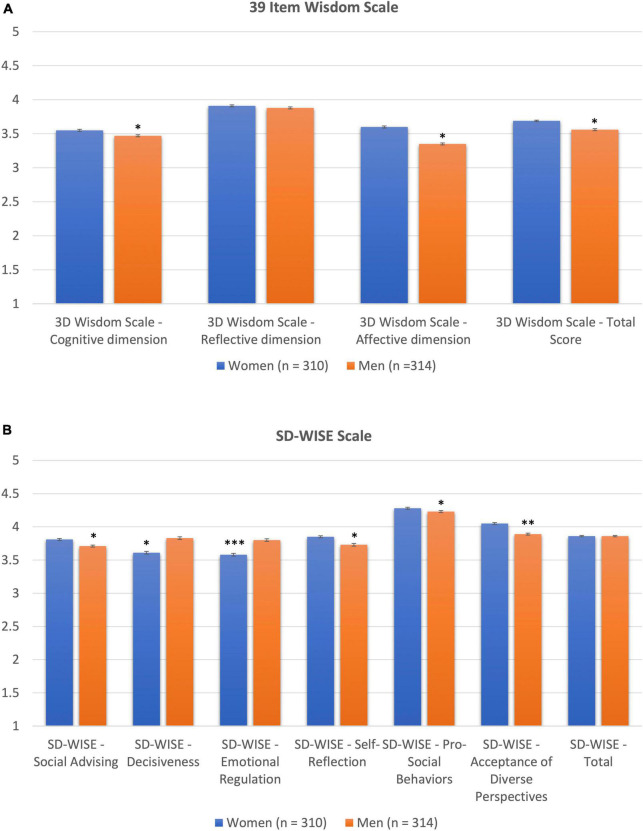
**(A)** Gender differences in 3D Wisdom Scores. **(B)** Gender differences in SD-WISE scores. *p < 0.05, ^**^p < 0.01, ^***^p < 0.001. Error bars represent standard error.

On the 3D-WS, the mean score among women was significantly higher on the Affective or Compassionate Dimension subscale score relative to men, *p* = 0.008 with a medium effect size (Cohen’s *d* = 0.481). Men scored significantly higher on the Cognitive Dimension, *p* = 0.019, with a small effect size (Cohen’s *d* = 0.184). Women also had higher 3D-WS total score, *p* = 0.01, with a small effect size (Cohen’s *d* = 0.292). Please see Figure 1A and Table 1.

There were significant differences between women and men in all six subscales of the SD-WISE (all *p* < 0.05, Figure 1B and Table 1). These differences varied in direction such that men had higher scores on Emotional Regulation and Decisiveness, while women had higher scores on Insight, Tolerance of Divergent Values, Pro-social Behaviors, and Social Advising. These differences had small-to-medium effect sizes (Cohen’s *d* = 0.110–0.331), with the Emotion Regulation effect being the largest. There was no significant difference between women and men in the overall SD-WISE score, indicating that the relative strengths and weaknesses of each group balanced out in the overall score.

Median splits created “high wisdom” and “low wisdom” categories for each wisdom measure and subdomain to identify whether there was variability in size of gender effects by group. For 3D-WS Reflective Dimension, people in the “low wisdom” category had a larger gender difference such that women scored higher in wisdom than men, *p* < 0.001 (η^2^*_*p*_* = 0.010). There was no significant difference between women and men in the “high wisdom” group for 3D-WS Reflective Dimension, *p* = 0.17. There were no other differences between “high wisdom” and “low wisdom” in any other categories.

Exploratory analyses examined how gender may moderate the relationship between wisdom and measures of well-being. Main effects supported the relationship between these variables and wisdom regardless of gender; however, gender did not moderate the relationship between wisdom and any of the measures of well-being including depression, loneliness, mental well-being, optimism, and resilience (Supplementary Tables 1–5).

## Discussion

This study on gender differences in wisdom found evidence that women and men differed on some components of wisdom. However, these differences were not uniform, but rather varied based on subdomains of wisdom as hypothesized. Women scored higher on several subdomains associated with social connection and compassion, including 3D-WS Affective or Compassionate Dimension, and SD-WISE Acceptance of Diverse Perspectives, Pro-Social Behavior and Social Advising subscales. We did not hypothesize differences in the reflection subdomains; women also scored higher on SD-WISE Insight subscale, and women in the lower half of the median split scored higher than men in the 3D-WS Reflective Dimension. On the other hand, men scored higher on SD-WISE and Emotional Regulation and Decisiveness subscales, and the Cognitive Dimension of the 3D-WS. Our third hypothesis was only confirmed by one of the wisdom measures: total wisdom scores were higher among women on the 3D-WS but not on the SD-WISE total score. SD-WISE has 6 components and offers a more detailed examination of wisdom, with relative strengths in each gender being neutralized by relative weaknesses in others. Only one measure, the 3D-WS Reflective, supported theory and evidence that there are larger gender differences among people with less wisdom. We also did not find any evidence that the gender moderated the relationship between wisdom and measures of well-being including depression, loneliness, mental well-being, optimism, and resilience.

Although some of these findings were unanticipated, others were well-aligned with past research in this and related areas. Acceptance of Diverse Perspectives and Social Advising both require perspective-taking and interest in the well-being and values of others even when they are misaligned with one’s own. It seems likely that both of these domains are related to compassion, a domain that women reliably score higher (Eisenberg and Lennon, 1983; Sprecher and Fehr, 2005; Martins et al., 2013). Previous research has found evidence that older men score higher on cognitive domains of wisdom (Ardelt, 2009), and that older women score lower on these domains (Cheraghi et al., 2015). Cheraghi and colleagues’ findings may have been significantly impacted by its setting in Iran, leading to much stronger differences than found in Ardelt’s (2009) study or our own. In our study, we found that men of all ages scored higher on cognitive domains of wisdom and Decisiveness in particular, a novel finding. Our findings that men scored higher on Emotion Regulation and women scored higher on one measure of Self-Reflection were also novel.

There are two potential causes for the gender differences we identified. One is biological. In this regard, it is important not to conflate sex and gender, but rather to discuss the potential impact of biological processes including sex on differences in wisdom. The finding of greater empathy and compassion toward others in women has been reported across time periods and across cultures. Sex-based differences in oxytocin receptor gene polymorphism may lead to increased empathy in women (Wu et al., 2012). In a different lab-based study, women and men performed similarly on three empathy tasks, but women activated more emotion- and self-based regions of the brain while men activated more cognitive-based regions, somewhat aligned with our findings (Derntl et al., 2010). In another study, women and men showed differences in neural activity during completion of emotion regulation tasks, although it was unclear whether these differences were innate and/or learned (McRae et al., 2008). This latter concept is important to consider; variance in biological processes can be externally caused, driven by sociocultural processes and the socialization that results. These findings, taken together, might indicate that individuals may use different strategies to achieve wisdom-associated goals based on biological and/or sociocultural processes, but that the strategy itself may not be particularly important as long as it works well for the individual and taps into at least one wisdom subcomponent. That is, it may not be necessary to have a high level of wisdom in all areas as long as the strengths an individual does have work effectively across aspects of that person’s life.

These sociocultural processes mentioned are the second, and likely to be the most influential cause for the observed gender difference in wisdom, including social expectations, gender norms and how wisdom-relevant behaviors are differentially reinforced between boys/men and girls/women by parents, teachers, peers, and society at large. Boys and men tend to be socialized toward behaviors including toughness and leadership, which may translate into being more decisive and in control of emotions, whereas girls and women tend to be socialized toward behaviors including warmth and caretaking, which may translate into pro-social behaviors including being compassionate and accepting of diverse people and ideas (Eagly, 2013). Male adolescents conceptualize wise people as somewhat more calculating, strict, and questioning than female adolescents do (Hollingworth et al., 2013); a description that fits well with being decisive and more focused on cognitive processes rather than emotion. Female adolescents conceptualize wise people as somewhat more cooperative, optimistic, extroverted, and spontaneous than male adolescents do (Hollingworth et al., 2013); which similarly aligns with being compassionate and oriented toward others. Additionally, men tend to report that work situations help develop wisdom, while women report that family-related and loss situations help develop wisdom (Glück et al., 2009). Furthermore, when people are asked to describe a wise man or a wise woman, wise women are somewhat more likely to be described as having compassion for others (Glück et al., 2009). Therefore, it appears that there are differences in how people conceptualize and actively develop wisdom by gender, indicating sociocultural causes based in gender norms. This may similarly indicate that women and men focus on different subdomains of wisdom as personal areas of growth to work toward, based on what they personally value, which is naturally situated within sociocultural context and gender norms. Of course, there is variance in gender norms by culture and region, and these norms change over time and by generation. This may explain why there is some variability in findings about gender differences in wisdom, given that research has been conducted in multiple different countries and regions with samples of different racial groups and age groups (e.g., Yang, 2001; Kanwar, 2013; Bang, 2015; Cheraghi et al., 2015; Maroof et al., 2015).

It has been argued that wisdom should be a broadly gender-neutral construct (Levenson, 2009) so that it does not facilitate gender bias by promoting attributes that current gender norms associate with one gender or another. Another theory (Orwoll and Achenbaum, 1993; Ardelt, 2009) posited that gender differences in wisdom may only be present at lower levels of wisdom, because very wise people will be strong in all wisdom subdomains. However, we found evidence for this in only one subdomain of wisdom, 3D-WS Reflective. Given our findings, a two-prong question can be considered: is the construct of wisdom gender neutral, and if it is, are we observing gender bias in current measures of wisdom? Our first comment regarding this question is that the route to wisdom does not have to be identical for each person- and indeed, we would argue that a society benefits from diversity in this respect, as in many other respects. Additionally, as observed in the emotion regulation and empathy literature (e.g., McRae et al., 2008; Derntl et al., 2010), individuals may use different strategies or subdomains of wisdom to achieve similar goals, indicating that there are many paths to a wise life, and high levels of wisdom in each subdomain is not necessary. For example, a woman and a man pursuing wisdom through individualized pathways might react to interpersonal conflict in different ways. The woman, with strengths in compassion, self-reflection, and acceptance of diverse perspectives, might seek to understand the conflict through the other person’s point of view, and show compassion for that person and their perspective even if they disagree. In contrast, the man, with strengths in emotional regulation and decisiveness, might retain emotional equilibrium despite the conflict and come to an understanding within himself that their relationship with that other person is more important than the issue they are disagreeing about, and therefore, decide to move beyond it instead of continuing the conflict. Therefore, the outcome may look similar from the outside – prioritizing personal relationship above the conflict -but the strategies used to achieve the outcome would be different.

Therefore, it seems to us that variance by subgroup in strengths and weaknesses at the wisdom subdomain level is not a flaw. It may not indicate gender bias as much as it does allowing for a diversity of paths toward wisdom. However, given the alignment between our findings and what has been previously noted to be valued as wise behavior by women and men, we wonder whether these differences might lessen as the divide in societal expectations of women and men fades. We would also note that these effects were small, indicating that although differences existed, women and men still had meaningful overlap as groups in their wisdom scores.

Finally, it seems that, at the macro level, the SD-WISE measure does not show evidence of an overall gender difference, unlike 3D-WS. The subcomponents included in a measure of wisdom will of course impact gender and other group differences. These two measures of wisdom measures were developed conceptually and tested psychometrically, using theoretical and empirical findings on layperson and expert definitions of wisdom without focusing on gender balance (c.f., Ardelt, 2003; Thomas et al., 2019; Jeste et al., 2020b,c). Variance in development choices (for example, separating out aspects of cognitive subcomponents of wisdom in the SD-WISE compared in combining them into one scale in the 3D-WS) may have influenced balance and therefore our findings. Given that wisdom is intended to be balanced, or without bias at least in its total score, measure developers, and researchers choosing measures, should consider these issues to avoid accidental bias. Glück et al.’s (2013) paper assessed five measures of wisdom, including the 3D-WS (the SD-WISE was developed later) and provided general recommendations to guide selection.

### Study Limitations

The cross-sectional design prevents assessment of differences in how wisdom develops and evolves over the lifespan. Future longitudinal work will be able to fully describe this development by gender, and the influence of other important factors including those associated with mental health and wellbeing. Longitudinal work may also be able to examine some of the hypotheses we and others have considered in regards to why and how gender differences occur. Participants self-identified their genders, and only binary options were offered so there were no options for people who are non-binary or other genders. Understanding wisdom profiles of non-binary people in future studies would be particularly enlightening in understanding how wisdom develops among people who may be less bound to traditional gender norms. We did not collect data to identify how many participants were transgender, which would aid in understanding whether transgender women and men have unique wisdom profiles relative to *cis* women and men. We did not collect any biomarkers relevant to gender or sex, so assessment of the potential impact of hormones or other factors was not possible. The study sample was predominantly white and came from an urban county in the United States; thus, the findings may not apply to other race/ethnic groups and different cultural regions, and study of other groups is important. We should point out, however, that in a recent study using SD-WISE and ULS scale for loneliness, we found that the constructs of wisdom and loneliness seemed to be largely similar in a San Diego sample of middle-aged and older adults and an age-comparable sample from rural Italy (Jeste et al., 2020a).

### Implications

On average, both women and men have strengths in wisdom subdomains that can be capitalized upon to promote their well-being. Helping people identify and lean on these strengths may promote related aspects of well-being including social connection and happiness. We also find that both groups have relative weaknesses that may benefit from individual and societal intervention to improve well-being and promote healthy living, including the growing set of positive psychiatry interventions. Consistent with past literature, we find a difference in compassion between women and men. There are a number of compassion interventions including compassion-focused therapy (Kirby et al., 2017). We also found that women scored higher in self-reflection than men. Engagement in most therapies promotes self-reflection and insight, making it a useful approach for those looking to strengthen these subdomains. Additionally, spiritual and non-therapy-based mindfulness practices may also promote insight. We found that men are higher in decisiveness than women. It is worth adding that the SD-WISE is unique in being validated as a wisdom scale with decisiveness as one of its major components. Intervention strategies like problem-solving therapy (Bell and D’Zurilla, 2009) may improve decisiveness and well-being. Similarly, this study is the first to report that men are higher in emotional regulation than women; mindfulness-based interventions including mindfulness-based stress reduction can improve emotion regulation (Guendelman et al., 2017). The finding of gender differences in components of wisdom suggests that assessing relative strengths and weaknesses in wisdom subdomains at the individual level may be helpful in guiding treatment planning across groups. However, our results did not find evidence that these varying wisdom profiles between women and men impacted the relationship between wisdom and other constructs typically targeted by positive psychiatry interventions, like resilience and optimism. One possible implication is that tailoring positive psychiatry intervention to individual wisdom profiles may be an effective strategy to improving these other, consistently associated constructs. Public health initiatives that target key subdomains among subgroups may be helpful to promote wellness.

## Data Availability Statement

The raw data supporting the conclusions of this article will be made available by the authors, without undue reservation.

## Ethics Statement

The studies involving human participants were reviewed and approved by the University of California, San Diego Human Research Protections Program. Written informed consent for participation was not required for this study in accordance with the national legislation and the institutional requirements.

## Author Contributions

ET, BP, EL, and DJ conceptualized the study. ET completed the initial analyses with conceptual support from DJ and conceptual and pragmatic support from T-CW, MT, and XT, wrote the first draft of the manuscript, and made the first draft of the tables and figures. RD provided database management and analytic support. All authors contributed to revisions of the manuscript, tables, and figures.

## Conflict of Interest

The authors declare that the research was conducted in the absence of any commercial or financial relationships that could be construed as a potential conflict of interest.

## Publisher’s Note

All claims expressed in this article are solely those of the authors and do not necessarily represent those of their affiliated organizations, or those of the publisher, the editors and the reviewers. Any product that may be evaluated in this article, or claim that may be made by its manufacturer, is not guaranteed or endorsed by the publisher.
