# Supplementary material for: Women and Men Differ in Relative Strengths in Wisdom Profiles: A Study of 659 Adults Across the Lifespan
Source: Front Psychol. 2022 Feb 3;12:769294. doi: 10.3389/fpsyg.2021.769294 (PMC8850272; doi:10.3389/fpsyg.2021.769294)
Supplement: Supplementary file 1 [file Data_Sheet_1.pdf]

Supplemental Table 1. Relationship between gender and wisdom and impact on Center of Epidemiologic Studies Depression Scale (CES-D; N = 659).

	**Model 1: 3DWS Cognitive**		**Model 2: 3DWS Reflective**		**Model 3: 3DWS Affective**		**Model 4: 3DWS Total**		**Model 5: SD-WISE Social Advising**		**Model 6: SD-WISE Decisiveness**		**Model 7: SD-WISE Emotional Regulation**		**Model 8: SD-WISE I Self-Reflection**		**Model 9: SD-WISE Pro-Social Behaviors**		**Model 10: SD-WISE Acceptance of Diverse Perspectives**		**Model 11: SD-WISE Total**	
Variable	**Est**	**SE**	**Est**	**SE**	**Est**	**SE**	**Est**	**SE**	**Est**	**SE**	**Est**	**SE**	**Est**	**SE**	**Est**	**SE**	**Est**	**SE**	**Est**	**SE**	**Est**	**SE**
Gender	.08	.70	-1.06	.93	.21	.86	-.19	.61	3.18	2.66	1.19	2.03	2.09	2.17	-1.46	2.48	-1.11	3.41	-2.55	2.79	-1.15	3.40
Wisdom	-.09	.09	-.42[a]	.15	-.13	.12	-.15	.08	-1.22	.52	-1.94[c]	.35	-2.86[c]	.39	-1.53[b]	.46	-3.54[c]	.56	-2.10[c]	.52	-4.53[c]	0.63
Gender by Wisdom	-.09	.18	.24	.23	-.12	.24	.02	.15	-.95	.70	-.26	.53	-.45	.58	.28	.64	.17	.80	.51	.69	.24	.87
R^2	.00		.01		.00		.01		.04		.09		.15		.03		.11		.04		.14	
F	.91		3.03[a]		.99		1.54		9.22[c]		20.5[c]		38.26[c]		6.39[c]		25.3[c]		9.58[c]		34.15[c]	

[a]p < .05, [b]p < .01, [c]p < .001. Coefficient p values adjusted using false discovery rate (FDR).

Supplemental Table 2. Relationship between gender and wisdom and impact on SF-Well-Being, Mental Component (N = 659).

	**Model 1: 3DWS Cognitive**		**Model 2: 3DWS Reflective**		**Model 3: 3DWS Affective**		**Model 4: 3DWS Total**		**Model 5: SD-WISE Social Advising**		**Model 6: SD-WISE Decisiveness**		**Model 7: SD-WISE Emotional Regulation**		**Model 8: SD-WISE Self-Reflection**		**Model 9: SD-WISE Pro-Social Behaviors**		**Model 10: SD-WISE Acceptance of Diverse Perspectives**		**Model 11: SD-WISE Total**	
Variable	**Est**	**SE**	**Est**	**SE**	**Est**	**SE**	**Est**	**SE**	**Est**	**SE**	**Est**	**SE**	**Est**	**SE**	**Est**	**SE**	**Est**	**SE**	**Est**	**SE**	**Est**	**SE**
Gender	1.19	1.17	.07	1.60	-1.00	1.44	1.13	1.03	-8.67	4.43	-3.54	3.34	-1.59	3.53	4.36	4.22	-1.34[c]	5.61	3.17	4.68	.80[c]	5.69
Wisdom	.09	.16	.38	.27	-.06	.21	.09	.14	1.43	.87	3.57[c]	.58	5.42[c]	.64	1.88	.79	5.73	.93	2.40[a]	.87	7.34	1.06
Gender by Wisdom	.00	.30	.29	.39	.68	.39	.02	.25	2.69	1.16	1.04	.87	.042	.94	-.79	1.09	.67	1.31	-.42	1.16	.11	1.46
R^2	.01		.02		.01		.01		.05		.12		.19		.02		.12		.03		.14	
F	1.23		3.46[a]		2.10		1.30		11.55[c]		30.34[c]		49.05[c]		3.55[a]		29.81[c]		5.73[c]		35.4[c]	

[a]p < .05, [b]p < .01, [c]p < .001. Coefficient p values adjusted using false discovery rate (FDR).

Supplemental Table 3. Relationship between gender and wisdom and impact on Life Orientation Task Revised (LOT-R; N = 659).

	**Model 1: 3DWS Cognitive**		**Model 2: 3DWS Reflective**		**Model 3: 3DWS Affective**		**Model 4: 3DWS Total**		**Model 5: SD-WISE Social Advising**		**Model 6: SD-WISE Decisiveness**		**Model 7: SD-WISE Emotional Regulation**		**Model 8: SD-WISE Self-Reflection**		**Model 9: SD-WISE Pro-Social Behaviors**		**Model 10: SD-WISE Acceptance of Diverse Perspectives**		**Model 11: SD-WISE Total**	
Variable	**Est**	**SE**	**Est**	**SE**	**Est**	**SE**	**Est**	**SE**	**Est**	**SE**	**Est**	**SE**	**Est**	**SE**	**Est**	**SE**	**Est**	**SE**	**Est**	**SE**	**Est**	**SE**
Gender	.72	.91	1.85	1.22	1.94	1.13	1.37	.80	7.09[c]	3.41	3.83[c]	2.64	2.69	2.89	7.67	3.20	-1.27	4.53	1.98[a]	3.66	9.86	4.45
Wisdom	.23	.12	.73[c]	.19	.48[a]	.16	.38[b]	.10	3.96	.67	2.94[c]	.45	3.83	.52	3.19[c]	.59	3.15[c]	.75	2.09	.68	6.76[c]	.82
Gender by Wisdom	-.06	.24	-.34	.30	-.43	.31	-.32	.19	-1.64	.90	-1.01	.69	-.77	.77	-1.79	.83	.49	1.06	-.26	.92	-2.39	1.14
R^2	.01		.03		.01		.02		.07		.08		.12		.05		.06		.03		.13	
F	1.84		6.25[c]		3.22[a]		4.89[b]		17.24[c]		19.23[c]		28.38[c]		12.02[c]		14.23[c]		6.65[c]		33.13[c]	

[a]p < .05, [b]p < .01, [c]p < .001. Coefficient p values adjusted using false discovery rate (FDR).

Supplemental Table 4. Relationship between gender and wisdom and impact on Connor-Davidson Resilience Scale (CD-RISC; N = 659).

	**Model 1: 3DWS Cognitive**		**Model 2: 3DWS Reflective**		**Model 3: 3DWS Affective**		**Model 4: 3DWS Total**		**Model 5: SD-WISE Social Advising**		**Model 6: SD-WISE Decisiveness**		**Model 7: SD-WISE Emotional Regulation**		**Model 8: SD-WISE Self-Reflection**		**Model 9: SD-WISE Pro-Social Behaviors**		**Model 10: SD-WISE Acceptance of Diverse Perspectives**		**Model 11: SD-WISE Total**	
Variable	**Est**	**SE**	**Est**	**SE**	**Est**	**SE**	**Est**	**SE**	**Est**	**SE**	**Est**	**SE**	**Est**	**SE**	**Est**	**SE**	**Est**	**SE**	**Est**	**SE**	**Est**	**SE**
Gender	1.54	1.02	1.83	1.36	.03	1.27	1.60	.90	-.88	3.64	-1.68	2.85	-3.41	3.03	2.40	3.54	-6.01	4.92	4.83	3.95	.73	4.56
Wisdom	.27	.13	.22[c]	3.82	.13	.18	.25	.12	4.98[c]	.71	3.77[c]	.49	5.11[c]	.55	3.57[c]	.66	4.57[c]	.81	4.69[c]	.73	9.21[c]	.84
Gender by Wisdom	-.09	.27	.33	-.44	.38	.35	-.14	.22	.74	.96	.58	.75	.96	.81	-.16	.92	1.81	1.15	-.69	.98	.16	1.17
R^2	.01		.04		.01		.01		.17		.16		.23		.09		.13		.11		.28	
F	3.34[a]		8.98[c]		2.60		3.06[a]		45.25[c]		41.66[c]		66.56[c]		21.09[c]		33.09[c]		28.01[c]		86.19[c]	

[a]p < .05, [b]p < .01, [c]p < .001. Coefficient p values adjusted using false discovery rate (FDR).

Supplemental Table 5. Relationship between gender and wisdom and impact on UCLA Loneliness Scale (ULS; N = 659).

	Model 1: 3DWS Cognitive		Model 2: 3DWS Reflective		Model 3: 3DWS Affective		Model 4: 3DWS Total		Model 5: SD-WISE Social Advising		Model 6: SD-WISE Decisiveness		Model 7: SD-WISE Emotional Regulation		Model 8: SD-WISE Self-Reflection		Model 9: SD-WISE Pro-Social Behaviors		Model 10: SD-WISE Acceptance of Diverse Perspectives		Model 11: SD-WISE Total	
Variable	**Est**	**SE**	**Est**	**SE**	**Est**	**SE**	**Est**	**SE**	**Est**	**SE**	**Est**	**SE**	**Est**	**SE**	**Est**	**SE**	**Est**	**SE**	**Est**	**SE**	**Est**	**SE**
Gender	.26	1.82	3.92	2.45	1.70	2.26	.92	1.59	10.10	6.89	13.49^{a}	5.24	5.53	5.85	3.44	6.49	2.13	8.62	-3.32	7.26	6.39	8.89
Wisdom	.15	.24	.36	.39	.63	.33	.45	.21	-3.96^{a}	1.35	-4.09^{c}	.89	-5.64^{c}	1.06	-2.70	1.21	-10.65^{c}	1.43	-5.44^{c}	1.34	-10.56^{c}	1.64
Gender by Wisdom	.29	.47	-.68	.60	-.11	.62	.07	.39	-2.47	1.81	-2.94	1.37	-.78	1.56	-.65	1.69	-.33	2.01	.96	1.81	-1.31	2.29
R^2	.13		.00		.01		.01		.05		.09		.08		.02		.15		.05		.13	
F	32.87^{c}		1.03		2.07		2.94^{a}		12.9^{c}		22.66^{c}		20.5^{c}		4.87^{b}		39.12^{c}		10.55^{c}		32.87 ^{c}	

$^{a}p < .05$, $^{b}p < .01$, $^{c}p < .001$. Coefficient p values adjusted using false discovery rate (FDR).
